# Acceptability of supporting lay-carer administration of anticipatory subcutaneous medications at home: a qualitative study using the theoretical framework of acceptability

**DOI:** 10.1186/s12904-025-01942-9

**Published:** 2025-11-24

**Authors:** David Sunkersing, Leila Shepherd, Calandra Feather, Ivor Williams, Imogen Eastwood, Joanne Droney, Bryony Dean Franklin, Bee Wee, Lisa O’Hara, Ara Darzi, Kate Grailey

**Affiliations:** 1 Imperial College London, London, UK; 2 Central and North West London NHS Foundation Trust, London, UK; 3 The Royal Marsden NHS Foundation Trust, London, UK; 4 Imperial College Healthcare NHS Trust, London, UK; UCL School of Pharmacy, London, UK; 5 Oxford University Hospitals NHS Foundation Trust, Oxford, UK; 6 Sussex Community NHS Foundation Trust, Sussex, UK

**Keywords:** Palliative Care, Qualitative Research, Person centred care, Human centred design, Innovation

## Abstract

**Background:**

Globally, evidence indicates that most people prefer to receive care and die at home, provided high-quality care is available. However, systemic and logistical challenges often prevent this outcome. Palliate is a nurse-led intervention aiming to address these barriers, supporting lay-carers in administering end-of-life subcutaneous medications to their loved ones, through training, written guidance, and documentation.

**Aim:**

To explore the perceptions and experiences of patients, carers, and healthcare professionals regarding the acceptability of the Palliate intervention, using the Theoretical Framework of Acceptability (TFA), including perceived barriers and opportunities to its implementation.

**Methods:**

A qualitative study was conducted using semi-structured interviews with healthcare professionals, patients, carers, and policy-makers, informed by the TFA. Data were analysed thematically using deductive analysis.

**Results:**

Thirty participants, including people with a diagnosis of advanced illness, carers, and a range of healthcare professionals involved in end-of-life care, provided perspectives on the acceptability of the intervention in end-of-life care. Participants described potential benefits, including improved symptom management, reduced waiting times for medication, and increased empowerment for families to support care at home. Concerns were raised about carer burden, emotional responsibility, and the need for professional oversight. Some participants spoke from direct experience of administering or supporting the intervention in practice, providing insights into both its practical value and the challenges of implementation.

**Conclusion:**

While the Palliate intervention was generally viewed as acceptable and potentially beneficial, its broader implementation requires careful consideration. Its acceptability was conditional on carers receiving clear training, ongoing professional support, and being able to participate voluntarily. These findings offer new insights into the boundaries of lay caregiving and have implications for the implementation of family-administered end-of-life care within health systems. Further research is needed to evaluate its safety, impact, and feasibility in diverse contexts before wider adoption can be recommended.

**Supplementary Information:**

The online version contains supplementary material available at 10.1186/s12904-025-01942-9.

## Introduction

In recent decades, end-of-life care policy has increasingly prioritised enabling people to die in their preferred place, most commonly their own home [1–3]. This policy direction reflects not only the preferences of many patients and families [4, 5], but also broader health system goals of reducing avoidable hospital admissions and improving the quality of palliative care [6–8].

An umbrella review found that, globally, home is the most preferred place of end-of-life care for both patients and family members and the most preferred place of death (51–55% in meta-analyses) [9]. However, despite strong preferences for a home death, actual place of death often differs, with congruence ranging from 21 to 100% [9]. Many people die in hospital instead, facing avoidable distress, unwanted interventions, and, for some, higher costs [10–14].

One recognised barrier to achieving a home-based death is the timely administration of symptom-relieving medications (e.g., for pain, nausea and other symptoms), particularly in the final days or hours of life. These medications commonly require subcutaneous or intravenous administration, as many people at the end-of-life cannot tolerate oral medications [15, 16]. At home, subcutaneous administration is often the most practical as it does not require invasive intravenous access [17]. Traditionally, subcutaneous drug administration in a patient’s home is carried out by a district or community nurse [18]. However, variation in resources, geographical location, and out-of-hours provision often results in delays and reduced access to timely care and medication availability – all of which can impact negatively on patient’s quality of life and end-of-life care [19–21].

Many strategies have been put forward to address this issue [15, 16, 22, 23]. In the UK, for example, a growing number of NHS Trusts have explored interventions that enable trained lay-carers to administer ‘as required’ (PRN) subcutaneous medications in the home setting [24, 25]. These interventions aim to facilitate faster symptom relief, reduce distress for patients and carers, and minimise the need for unscheduled admissions or professional call-outs [26, 27].

Evidence, particularly from Australia, has demonstrated that carer administration of subcutaneous medications at the end-of-life can be feasible, acceptable, and beneficial under appropriate conditions [28–30]. In the UK, interest in such approaches is growing [31–33], and some Trusts have developed policies and frameworks to operationalise this model [34, 35]. However, national implementation remains limited, and evidence regarding the acceptability, safety, and potential outcomes of these interventions in the UK context is still emerging [31].

The Palliate intervention (hereafter ‘Palliate’) is one such initiative, based on an existing model of lay-carer administered palliative care in Wales [36]. Palliate is a nurse-led, structured intervention including training, written guidance, and documentation to support lay-carers in administering anticipatory subcutaneous medications to their loved ones at the end-of-life, currently being integrated within a large London NHS Foundation Trust.

This study responds directly to two of the highest priorities for palliative and end-of-life care identified by the James Lind Alliance Priority Setting Partnership [37]: (1) how best to provide palliative care outside of normal working hours to prevent crises and support patients to remain in their preferred place of care, and (2) what information and training carers and families need to provide safe and effective care at home, including the administration of medicines. Using this context, the study investigates the barriers and opportunities associated with Palliate. Through engagement with patients, carers, and healthcare professionals, the research will identify outcomes that matter most to stakeholders, informing both the refinement of the intervention and the design of a future definitive clinical trial.

## Methods

### Study design

This study employed a qualitative, exploratory design, using semi-structured interviews to gather in-depth insights from a range of stakeholders. The approach was underpinned by the Theoretical Framework of Acceptability (TFA), which served as both a conceptual and an analytic guide [38, 39]. It provides a structured, theory-informed approach to understanding how acceptable the intervention was perceived to be by those delivering or receiving it.

The TFA is a framework that comprises seven key constructs: affective attitude, burden, ethicality, intervention coherence, opportunity costs, perceived effectiveness, and self-efficacy [39]. These domains helped shape both the data collection instruments and the analysis process.

Using the TFA ensured conceptual consistency across data collection and analysis, enabling systematic exploration of key dimensions of acceptability while also allowing for inductive insights beyond the predefined domains.

The explicit research question guiding this study was:

What are the perceptions and experiences of patients, caregivers, and healthcare professionals regarding the acceptability of lay-carer administration of anticipatory subcutaneous medications at home?

This study was carried out and is reported in line with COREQ guidance COREQ (COnsolidated criteria for REporting Qualitative research) Checklist [40].

### Participant sampling and recruitment

As part of the study design, participants were purposively sampled from four key stakeholder groups: Healthcare Professionals, Service Leaders and Policy Stakeholders, Informal Carers and Patients. Recruitment was planned for approximately 3–5 participants per group (around 25–30 in total), with the aim of including participants whose roles reflect those commonly involved in end-of-life care, based on NHS descriptions [41]. In line with qualitative research principles [42, 43], recruitment was reviewed throughout the study, with final numbers determined by data richness and the point at which no new concepts were emerging (i.e. thematic saturation).

Recruitment was carried out in the UK via existing networks, targeted outreach on social media (e.g., Twitter, Facebook, LinkedIn), and online platforms related to palliative care communities of practice. At one site delivering the Palliate intervention, a clinical specialist nurse affiliated with a regional NHS research network also helped identify and invite participants. Individuals with or without prior knowledge of the Palliate intervention were eligible, to capture both informed and ‘new’ perspectives. Participants had to be aged 18 or over and able to communicate in English.

### Data collection

A semi-structured topic guide was developed by the research team with reference to the TFA to ensure alignment with core constructs such as affective attitude, burden, and perceived effectiveness. The guide was designed to promote consistency across interviews while allowing flexibility to explore emergent themes as they arose in participant responses. It covered key areas including participants’ initial responses to the intervention concept, perceived benefits and challenges, anticipated emotional or practical burdens, and contextual factors influencing acceptability (Appendices 1–3).

Interviews were conducted online using Microsoft Teams and audio-recorded with informed consent. Demographic information was collected through a brief online questionnaire (administered via Qualtrics) [44] and summarised descriptively. Data collection continued until thematic saturation was achieved.

### Data analysis

Audio recordings were transcribed verbatim and anonymised. Transcripts were imported into NVivo 15 [45] for coding and analysis. A deductive thematic framework approach was used, guided by the constructs of the TFA, to evaluate the acceptability of the intervention in a structured and theory-informed manner. Transcripts were initially coded using a framework based on the seven TFA constructs (affective attitude, burden, ethicality, intervention coherence, opportunity costs, perceived effectiveness, and self-efficacy). While the primary approach was deductive, the coding process remained open to inductive insights to capture further unexpected but relevant nuances within TFA domains. Age was collected in categorical ranges. For the purposes of calculating the mean and standard deviation (SD), the midpoint of each range was used to estimate participants’ ages. In line with a deductive framework approach, the seven TFA constructs were treated as the primary analytic themes; we did not generate additional sub-themes. Variation within each domain (barriers and facilitators) is presented narratively with illustrative quotations, with a consolidated summary provided in Appendix 1, Table A1. Themes were refined through iterative team discussions, and discrepancies in coding were resolved by consensus. To enhance credibility and reduce potential bias, initial analyses were reviewed by a second researcher. A subset of transcripts was double-coded, with any differences collaboratively resolved to ensure consistency in applying the framework.

### Rigor and trustworthiness

To ensure rigour, the study followed key quality principles for qualitative research, including reflexivity, audit trail maintenance, and iterative theme checking [46, 47].

To ensure the trustworthiness of the study, we applied Guba and Lincoln’s four criteria of qualitative rigor. Credibility was enhanced using multiple coders, diverse participant representation, and iterative team discussions to confirm interpretations. Dependability was supported by maintaining an audit trail documenting methodological and analytic decisions, including coding iterations, theme development, and reflexive notes. The audit trail was reviewed by members of the research team and discussed with members of a study steering group to enhance transparency and consistency of the analytic process. Confirmability was reinforced through peer debriefing and ongoing team reflexivity to mitigate bias and ensure transparency. Transferability was supported through contextual detail and transparent reporting to facilitate comparison across settings.

### Researcher characteristics and reflexivity

The research team comprised a multidisciplinary group including researchers with expertise in public health, nurses with direct palliative care experience, palliative care physicians, and academics specialising in end-of-life care. This breadth of knowledge enabled rich collaboration and brought diverse clinical, public health, and academic perspectives to the study. Researchers involved in conducting and analysing the interviews were mindful of potential bias stemming from their professional backgrounds. To mitigate this, structured interview guides were used to ensure consistency, and the team engaged in ongoing discussions and reflexive practices throughout thematic analysis.

## Results

Thirty participants, including patients, lay carers, pharmacists, nurses, general practitioners (GPs), commissioners, and clinical leaders participated in a qualitative interview (Table 1) between October 2024 and April 2025. Participant demographics are summarised in Table 2.

**Table 1:** Key stakeholder groups

Key Stakeholder Group	Included within Stakeholder Group	Number of Participants
Healthcare Professionals	Palliative Care/End-of-Life Care Nurses	3
	District Nurses	3
	General Practitioners (GPs)	3
	Palliative Care/End-of-Life Care Doctors	2
	Non-Medical Prescribers (Pharmacists)	3
Service Leaders and Policy Stakeholders	Former National Clinical Director of a Palliative/End-of-Life Care Charity, National Director (Palliative/End-of-Life Care Charity), National Clinical Lead for End-of-Life care, Public Health Professional, ICB commissioner	5
Informal Carers	Lay carers with direct experience of using Palliate	3
	Lay carers without experience of using Palliate	5
Patients	Persons with a diagnosis of advanced illness (all without experience of Palliate)	3

**Table 2:** Participant demographics

Characteristics	Healthcare Professionals, Service Leaders and Policy Stakeholders	Informal Carers and Patients
Gender, *n* (%)		
Male	5 (26)	5 (45)
Female	14 (74)	6 (55)
Age (Years)		
Mean (SD)	52 (11)	58 (17)
Ethnicity, *n* (%)		
White – British	18 (95)	10 (91)
Asian or Asian British – Indian	1 (5)	1 (9)
Education level, *n* (%)	Undergraduate or Postgraduate degree: 19 (100)	*(Informal Carers)* Undergraduate or Postgraduate degree: 7 (87.5) Vocational/technical training: 1 (12.5) *(Patients)* Undergraduate or Postgraduate degree: 2 (66.6) A-Levels, or equivalent: 1 (33.3)
Job position/role, *n* (%)	Palliative care/End-of-life nurses: 3 (15.8); District nurses: 3 (15.8); GPs: 3 (15.8); Palliative care doctors: 2 (10.5); Pharmacists: 3 (15.8); Service leaders and policy stakeholders: 5 (26.3)	Patients 3 (27); Lay carers 8 (73)
Work experience in end-of-life care (years)	Mean (SD): 18 (8.4)	-
Primary diagnosis (*patients*), *n* (%)	-	Advanced cancer 3 (100)
Relationship to patient (*informal carers only*), *n* (%)	-	Spouse/partner: 3 (37.5); Adult child/grandchild: 4 (50); Friend: 1 (12.5)

Findings are presented in relation to the seven constructs of the TFA, with illustrative quotations provided to capture both perceived barriers and opportunities within each domain. Across all domains, participants expressed a balance of perceived facilitators of acceptability and identifiable barriers, which often reflected emotional, ethical, or practical concerns emerging within the structure of the TFA.

### Affective attitude

This construct refers to how participants felt about Palliate on an emotional level. Participants generally expressed positive attitudes towards the intervention, recognising its potential to empower carers and alleviate professional workload. One pharmacist reflected:

*“I think it’s a good idea actually. Really useful. Benefit a lot of people. It’s a really good step in the right direction and I think it’s long overdue.”* (Case 6, pharmacist).

Carers expressed a willingness to participate, emphasising the value of enabling loved ones to remain at home with dignity. One carer described:

*“Yeah. I liked everything about it. Because as I say*,* it resonates with me closely.”* (Case 15, lay carer, has experience of using Palliate).

However, some healthcare professionals highlighted mixed feelings, noting the potential emotional discomfort for laypeople tasked with administering end-of-life medication. As one district nurse explained:

*“Sort of like in the middle. Some people*,* absolutely*,* other people*,* I would have concerns. I’d want to know them well*,* and I’d want to see them do it. Yeah*,* I would be a little bit worried.”* (Case 10, district nurse).

This domain captured both enthusiasm and emotional ambivalence, revealing an underlying barrier of emotional discomfort that shaped the perceived acceptability of the intervention.

### Burden

This construct refers to the perceived effort required from participants to engage with Palliate. Both carers and professionals acknowledged the emotional and practical burden associated with Palliate. One carer described the intensity of the responsibility:

*“Physically and mentally and emotionally*,* it is draining*,* yes… just being… 24 hours a day*,* day after day… I gave her a lot of injections… like 70 or 80 injections over time.”* (Case 16, lay carer, has experience of using Palliate).

Some professionals argued that this initial investment of time and emotional effort could reduce overall service demand. A GP remarked:

*“It was a little bit time heavy at the start to make sure that the relatives felt confident and sufficiently trained… but then once it was all in place*,* it saves on at least a number of clinical contact points.”* (Case 12, GP, involved in recommending Palliate).

Yet, concerns about emotional overwhelm persisted particularly in relation to the emotional demands placed on both clinicians and families during complex decision-making processes. One palliative care doctor reflected:

*“We underestimate the terror of doing that… do we underestimate the power we are investing in families?”* (Case 19, former national clinical director of a palliative/end-of-life care charity – in reference to medication administration).

Within this domain, burden was expressed primarily through emotional strain, perceived role overload, and the weight of responsibility, highlighting barriers related to psychological and workload challenges.

### Ethicality

This construct refers to how well Palliate aligned with participants’ personal values or professional principles. Participants raised ethical questions about shifting responsibility to family members. A pharmacist highlighted the risk of role conflict:

*“Are we putting people in a difficult position because we’re making them carers rather than family? That’s why lay care administration is always something that should be voluntary and very*,* very willing.”* (Case 6, pharmacist).

Carers similarly expressed apprehension about the possibility of causing harm. One patient shared:

*“Fear of hurting the patient and what happens if I cause them harm in any way. There might be some people who think*,* well actually*,* why isn’t a medical professional who is trained for this doing it?”* (Case 5, patient).

Nevertheless, some participants viewed Palliate as an act of compassion. One carer reflected:

*“It certainly didn’t feel like there’s any kind of*,* you know*,* moral or ethical issues for me. You know*,* I’m giving care to my dying wife. Seems like a very compassionate thing to do.”* (Case 16, lay carer, has experience of using Palliate).

This domain revealed ethical tensions as key barriers, particularly concerns about role conflict, moral discomfort, and perceived inequities in the distribution of responsibility. This was contrasted with views that framed the practice as compassionate and aligned with personal values.

### Perceived effectiveness

This construct refers to the extent to which participants believed Palliate would achieve its intended outcomes. Many participants believed Palliate could significantly improve symptom management and reduce unnecessary distress caused by waiting for professional attendance. A district nurse commented:

*“I’ve seen it where we’ve turned up to patients late and they’re in agony. And if they’d had somebody else there to give them that*,* it would definitely mean that people wouldn’t necessarily get to that level.”* (Case 10, district nurse).

Similarly, a carer emphasised the benefit of immediate access to relief:

*[Participant had been asked whether they felt the intervention helped manage their loved one’s symptoms]: “Very big yes… given the extreme level of pain she was in and how distressing it was and the importance of being at home… there’s a very big yes to that question.”* (Case 16, lay carer, has experience of using Palliate).

However, several participants noted that success would depend on proper carer selection and adequate support. As one palliative care doctor highlighted:

*“If you choose your patient and your family carefully*,* I think they will have a better ending… they’re likely to get more timely symptom control.”* (Case 19, former national clinical director of a palliative/end-of-life care charity).

*[Participant had been asked whether they felt the intervention would improve the management of patient symptoms at home]*: *“I think unreservedly*,* yes… anything that… tested as successful and effective… that can improve dying at home can only be a good thing.”* (Case 20, public health professional).

Barriers within this domain were linked to concerns about inconsistent implementation, variability in carer capability, and dependence on robust support systems, which were perceived as prerequisites for effectiveness.

### Intervention coherence

This construct refers to how well participants understood Palliate and its purpose. Most participants demonstrated a clear conceptual understanding of Palliate. A commissioner stated:

*“This one is really a no-brainer. You can explain it in two sentences and it’s just really obvious what it is.”* (Case 7, ICB commissioner, has experience commissioning Palliate in their region).

One patient further explained the straightforward nature of the process:

*“Administering meds under the skin is fairly simple. It’s something that’s done in some community settings by carers*,* and so that is quite feasible to train somebody because it’s quite a simple process.”* (Case 11, patient).

Suggestions for improving coherence included the use of pre-measured doses and video-based training, as noted by a public health professional:

*“I think backing the training up with videos is a great idea… that would make life much easier*,* particularly for older people.”* (Case 20, public health professional).

Here, barriers emerged around informational clarity and confidence in training resources. Participants’ suggestions for structured, accessible materials indicated an opportunity to enhance comprehension and consistency of understanding.

### Self-Efficacy

This construct refers to participants’ confidence in their ability to successfully engage with and carry out Palliate. While several participants felt that carers could develop the required skills with adequate training, confidence levels varied. A pharmacist noted rapid competence in one case:

*“[Talking about a carer she trained]: She picked it up in a couple of hours*,* was happy*,* comfortable*,* competent within a couple of hours of training.”* (Case 1, pharmacist, has experience of using a variation of Palliate).

A patient expressed conditional confidence:

*“I think that as long as they’re trained to do it*,* then that yeah*,* that’s fine. If it’s something they feel that they could do*,* then good. But if not*,* then maybe not everybody would feel comfortable doing it.”* (Case 2, patient).

However, some participants highlighted the anxiety that carers might feel when performing clinical tasks:

*“I would worry about them being scared about doing that… the amount of people that say to [me]*,* ‘I’m not a nurse. I don’t know. I’m not a nurse.’”* (Case 26, end-of-life care nurse).

This domain expressed barriers in terms of variable confidence and fear of clinical responsibility, underscoring the need for sustained reassurance, supervision, and training reinforcement.

### Opportunity costs

This construct refers to what participants felt they might have to give up (e.g., time, resources, other activities) to engage with Palliate. Carers reflected on the significant personal sacrifices required. One carer described the total immersion in the caregiving role:

*“I gave up everything else and just looked after [name] … that was my new job.”* (Case 13, lay carer, has experience of using Palliate).

Professionals acknowledged these challenges, particularly for those with other commitments:

*“It’s mainly going to be suited to carers who are there all the time. Otherwise it’s pretty pointless teaching somebody who’s not going to be there when there’s a crisis.”* (Case 19, former national clinical director of a palliative/end-of-life care charity).

Barriers within this domain were expressed through the high personal cost of time, emotional energy, and social withdrawal, suggesting that acceptability was conditional on adequate support and flexibility in carer involvement.

### General acceptability

Despite identified challenges, participants largely viewed Palliate as acceptable and beneficial. One carer reflected on the positive impact:

*“It was invaluable… I think it would have been a very different*,* much worse experience to have gone through what we went through without this… I felt very privileged and grateful to be part of it.”* (Case 16, lay carer, has experience of using Palliate).

Healthcare professionals similarly expressed general support, provided appropriate safeguards and training were in place. One palliative care doctor stated:

*“Yes*,* I think they’re very acceptable… anything to support patients and families to feel empowered*,* remain at home*,* and have good symptom management.”* (Case 8, palliative care doctor).

Across the seven TFA domains, participants generally perceived Palliate as acceptable, provided carers were supported through appropriate training, safeguards, and voluntary participation. Barriers were expressed through emotional discomfort, ethical tensions, role burden, and perceived gaps in training or support, balanced by strong perceived benefits such as improved timeliness of care, empowerment of carers, and alignment with patient-centred values. Overall, this synthesis illustrates both the intervention’s perceived value and the contextual conditions shaping its acceptability. The TFA provided a useful lens for identifying these facilitators and barriers within each domain.

A consolidated summary table mapping barriers and facilitators to TFA domains, with verbatim quotations, is provided in Additional file 1.

## Discussion

This study explored stakeholder perspectives on Palliate, a lay-carer administered subcutaneous medication intervention designed to support end-of-life care at home. The findings provide early insights into how such models may be implemented within health systems increasingly oriented toward care at home. While the concept of family-administered subcutaneous medication is not new, structured, nurse-led interventions like Palliate remain underexplored in the literature. By engaging a broad range of stakeholders, including patients, carers, clinicians, and policy-makers, it offers a nuanced understanding of the practical, ethical, and emotional dimensions of lay-carer involvement in end-of-life medication administration, grounded in both hypothetical and real-world experiences.

Our findings reveal that while Palliate is generally perceived as acceptable and potentially beneficial, it presents a complex balance of opportunities and risks that require careful consideration.

The data suggest that, although participants across all groups recognised the intervention’s potential to improve timely symptom management and empower carers, concerns were raised about the emotional, ethical, and practical burdens placed on family members. Carers spoke of feeling privileged to help their loved ones, yet also acknowledged the toll such responsibility might take on their emotional wellbeing and daily life. Healthcare professionals echoed this tension, recognising both the practical advantages and the moral complexities of shifting clinical tasks to lay carers.

Notably, several participants in this study were not speaking hypothetically; some had direct experience of administering medication to a loved one, while others were clinicians who had already implemented or recommended the Palliate intervention in practice. These insights grounded the data in real-world experiences, adding ecological validity to the findings and highlighting both the practical benefits and emotional challenges of the intervention. While this blend of hypothetical and experiential accounts strengthens the study, it also underlines the variability of individual experiences, reinforcing the need for tailored implementation strategies.

This balancing act is consistent with existing research, which highlights that interventions designed to empower family carers at the end-of-life often carry unintended emotional consequences, including heightened feelings of responsibility, anxiety, and guilt [48, 49]. Moreover, this aligns with studies demonstrating that while carers value being involved, they frequently report feeling underprepared or unsupported when clinical responsibilities are delegated to them [50, 51].

Our findings also highlight the importance of context. This study was conducted within the UK’s National Health Service (NHS), where national policy increasingly promotes care closer to home [52]. In this context, Palliate represents an opportunity to support the shift towards more person-centred, community-based care, by aligning care delivery with patient preferences and national priorities. For example, this aligns with recommendations from the Commission on Palliative and End-of-Life Care, which emphasise shifting care from hospital to community [53]. In contrast, healthcare systems in other countries such as Australia and Canada may differ in how they resource palliative care services, with some models offering greater community-based support or regionalised interventions [54, 55]. As such, the feasibility and acceptability of family-administered interventions may vary internationally, influenced by differing service models, legal frameworks, and cultural expectations around family caregiving.

Importantly, several participants raised concerns about the potential conflation of this intervention with assisted dying, now, set to become legalised in England and Wales [56]. While Palliate is specifically designed to manage distressing symptoms at the end-of-life – not to accelerate death – such perceptions reflect the ethical sensitivity in which this intervention operates. This distinction must be explicitly communicated to families and professionals to safeguard against misunderstandings and ensure the intervention is perceived as a legitimate palliative care practice, distinct from assisted dying.

In addition to ethical considerations, the practical implications of storing injectable medications, particularly controlled drugs, within the home must be carefully addressed. This includes assessing the potential risks of misuse, accidental administration, or diversion, as well as the emotional burden placed on carers responsible for managing these medications. Clear guidance on safe storage, disposal procedures, and carer education will be essential components of any implementation strategy to ensure both safety and confidence in the use of the intervention at home.

In addition to these findings, the study contributes to theoretical understandings of acceptability in end-of-life care. Applying the TFA helped structure analysis across emotional, practical, and ethical domains. However, our findings suggest that in emotionally complex interventions like Palliate, constructs such as burden, ethicality, and opportunity costs are shaped not just by individual perceptions but by relational dynamics and systemic context. Acceptability was often conditional and potentially fluid, shifting with carers’ confidence, support structures, and evolving circumstances. These insights underscore both the utility of the TFA in structuring analysis and its limitations, as its predefined constructs may constrain the emergence of novel, cross-cutting themes. Future adaptation of the TFA might better capture the relational and contextual nuances of palliative care interventions.

The study gathered diverse perspectives from healthcare professionals and lay stakeholders, although ethnic diversity was limited. While the range of disciplines strengthens the relevance of findings, the limited ethnic diversity highlights the need for further research on how cultural beliefs and preferences might influence acceptability in more diverse populations.

### Implications for practice

Aligned with the TFA constructs of self-efficacy and burden, carers must be equipped with training that both builds confidence and avoids overwhelming them. Participants in this study emphasised the value of practical, hands-on demonstrations, supported by written or video resources tailored to individual learning needs. Training should be revisited over time to consolidate knowledge. International evidence supports this approach – for example, one study in Australia showed that lay carers can safely administer subcutaneous medications following structured, carer-centred training [29].

The construct of perceived effectiveness was strongly tied to access to ongoing professional support. Participants valued access to clinical advice, particularly when symptom control became complex or when decisions around medication were required. This aligns with findings from a review of out-of-hours palliative care telephone services, which demonstrated that access to timely clinical support reduced unplanned hospitalisations and improved carer confidence and satisfaction [57].

Ethicality also emerged as a key consideration. Carers reflected on the emotional and moral weight of participating in end-of-life care, particularly in administering injectable medications. Addressing these concerns openly and directly is vital.

Finally, the TFA construct of opportunity costs underscores the importance of ensuring that carer participation remains voluntary. Healthcare professionals should assess each carer’s capacity, willingness, and broader personal circumstances before involving them in the intervention. Alternative care pathways should be offered when participation is not feasible or desired.

By grounding implementation strategies in the TFA and drawing on international evidence, services can establish safe, consistent practices, including tailored training, accessible clinical support, and flexible pathways, while remaining responsive to carers’ emotional, ethical, and practical needs across diverse cultural and legal contexts.

### Limitations

This was an exploratory qualitative study designed to generate insights rather than generalisable conclusions. As such, uncertainty was inherent to the interpretive nature of the analysis, and findings should be understood as context-specific. While the study included a range of professional and lay perspectives, the sample was geographically limited to the UK, with most participants identifying as White British. This limits the transferability of findings to other cultural or health system contexts, particularly where caregiving practices or ethical norms differ.

Additionally, while the study benefits from including participants with direct experience of administering or overseeing the intervention, these insights were not universal across the sample. Some participants spoke purely in hypothetical terms. This mix of experiential and hypothetical accounts adds depth but also introduces variability in familiarity and emotional proximity to the intervention. Participation bias may have also influenced the findings, as individuals with stronger views or experiences were potentially more likely to take part.

Reflexivity was embedded throughout the research process, with ongoing discussions among the multidisciplinary team to identify and mitigate potential bias during data collection and analysis. However, the researchers’ professional backgrounds in palliative and end-of-life care may have influenced the interpretation of findings.

Finally, the use of a deductive framework analysis guided by the TFA provided conceptual structure but may have constrained the emergence of novel or cross-cutting themes. Future research should explore alternative or complementary analytic approaches and evaluate larger-scale, real-world implementation to assess the broader applicability of findings.

### Opportunities for future work

Palliate is an example of a complex intervention, with multiple factors that are likely to contribute to successful implementation and delivery. The effectiveness of Palliate on outcomes that are important to patients needs to be tested through mixed-methods, multi-centre studies, with consideration of the impact of social determinants of health on outcomes and access. A deeper understanding of factors impacting successful and safe implementation, as well as robust health economic evaluation will help inform equitable delivery and business case planning. Finally, exploring the use of digital tools (e.g., telehealth check-ins, electronic medication records, and decision-support systems) offers an opportunity to enhance professional oversight while maintaining the autonomy and responsiveness that carers value.

## Conclusion

This study found Palliate to be broadly acceptable to a range of UK-based stakeholders, including patients, carers, and healthcare professionals. Participants highlighted potential benefits such as improved symptom management, faster access to medication, and greater autonomy for carers. However, they also identified significant emotional and ethical considerations, particularly around perceived responsibility, professional oversight, and the need to distinguish the intervention clearly from assisted dying.

Importantly, the findings suggest that acceptability is not fixed but conditional – shaped by interpersonal dynamics, emotional readiness, and system-level support. This study contributes novel insights into the relational and contextual factors influencing intervention acceptability, extending the application of the TFA in end-of-life care settings.

Implementation must be underpinned by clear training, ongoing professional support, and a voluntary model of participation that respects carers’ preferences and limits. Further research is needed to evaluate the intervention’s safety, effectiveness, and feasibility in diverse cultural and health system contexts, ensuring that lay carers are empowered rather than overburdened.

## Supplementary Information

Supplementary Material 1.

Supplementary Material 2.

## Data Availability

Data can be made available upon reasonable request from the corresponding author.
