# Supplementary material for: Acceptability of supporting lay-carer administration of anticipatory subcutaneous medications at home: a qualitative study using the theoretical framework of acceptability
Source: BMC Palliat Care. 2025 Nov 24;24:312. doi: 10.1186/s12904-025-01942-9 (PMC12750559; doi:10.1186/s12904-025-01942-9)
Supplement: Supplementary file 1 — Supplementary Material 1. [file 12904_2025_1942_MOESM1_ESM.docx]

**Appendices**

**Appendix 1: Semi-Structured Topic Guide: Patients**

Questions:

- Can you tell me a little about your experience and understanding of the intervention?
- What do you think of the intervention in general? (Opening question/ TFA - Affective attitude)

Prompt: *Do you like it, not like it? Why/why not?*

*Would you/are you comfortable with the idea of your friend/relative preparing and administering the medications for you?*

- Do you think as well as any other caring responsibilities, your [carer] taking on this task is reasonable? (TFA - Burden/intervention coherence)
- Do you feel there are any moral or ethical consequences to your *[carer] using this intervention to manage your symptoms at home? (TFA - Ethicality)

If clarification required: Ethical considerations are the things you think about when deciding whether something is right or wrong, based on what you believe is the right way to behave.

- How confident do/would you feel about a non-professional, i.e. your [carer] preparing and administering the medications for you? (TFA - Self-efficacy)
- Do you feel that the intervention helped/will help you to manage your symptoms at home? (TFA - Perceived effectiveness)

Expand if necessary – to describe specific end of life symptoms. (Refer back to PIS)

- Do you feel that there any aspects of using this intervention that might interfered with/get in the way of other priorities? (TFA - Opportunity costs)
- We’re coming to an end of the interview now. Overall how acceptable is this intervention/opportunity to you? (TFA - General acceptability)

**Appendix 2: Semi-Structured Topic Guide: Carers**

Questions:

- Can you tell me a little about your experience and understanding of the intervention?
- What do you think of the intervention in general? (Opening question/ TFA - Affective attitude)

Prompt: *Did you like it, not like it? Why/why not?*

*Where you comfortable preparing and administering the medications for .. (name of loved one).*

- Did you find the training with the nurse and the materials easy to follow? (TFA - Burden/intervention coherence)
- Do you feel there are any moral or ethical consequences to you using this intervention? (TFA - Ethicality)
- How confident did you feel when using the instructions to prepare and administer the medications on your own? (TFA - Self-efficacy)
- Did you feel that the intervention helped you to manage ..(name of loved one) symptoms at home? (TFA - Perceived effectiveness)
- Were there any aspects of using the intervention that interfered with/got in the way of other priorities? (TFA - Opportunity costs)
- We’re coming to an end of the interview now. Overall how acceptable was/is this intervention/opportunity to you? (TFA - General acceptability)

**Appendix 3: Semi-Structured Topic Guide: Professionals**

Questions:

- Can you tell me a little about your experience and understanding of the intervention?
- What do you think of the intervention in general? (Opening question/ TFA - Affective attitude)

Prompt: *Did you like it, not like it? Why/why not?*

- How much time and effort does it take to support carers using the Palliate intervention? (TFA - Burden)
- Do you find the training materials easy to use and follow? (TFA - Intervention coherence)
- Do you feel there are any moral or ethical consequences to you using the Palliate intervention? (TFA - Ethicality)
- How confident do you feel when using the Palliate intervention to support your patients and their carers? (TFA - Self-efficacy)
- Do you feel that the intervention has improved the management of patients’ symptoms at home? (TFA - Perceived effectiveness)
- Were there any aspects of using the Palliate intervention that interfere with/get in the way of other priorities? (TFA- Opportunity costs)
- We’re coming to an end of the interview now, overall how acceptable is the Palliate intervention/opportunity to you? (TFA - General acceptability)
