# Supplementary material for: Acceptability of supporting lay-carer administration of anticipatory subcutaneous medications at home: a qualitative study using the theoretical framework of acceptability
Source: BMC Palliat Care. 2025 Nov 24;24:312. doi: 10.1186/s12904-025-01942-9 (PMC12750559; doi:10.1186/s12904-025-01942-9)
Supplement: Supplementary file 2 — Supplementary Material 2. [file 12904_2025_1942_MOESM2_ESM.docx]

**Additional File 1**

**Table A1: Summary of findings mapped to the Theoretical Framework of Acceptability (TFA)**

| **TFA Domain** | **Description of Domain** | **Barriers** | **Facilitators / Enablers** | **Illustrative Quotation – Barrier** | **Illustrative Quotation – Facilitator** |
| --- | --- | --- | --- | --- | --- |
| **Affective Attitude** | Emotional responses to the intervention | Emotional discomfort; anxiety about giving medication | Positive attitudes; sense of helping loved ones | Sort of like in the middle. Some people, absolutely, other people, I would have concerns. I’d want to know them well, and I’d want to see them do it. Yeah, I would be a little bit worried. (Case 10, district nurse) | I think it's a good idea actually. Really useful. Benefit a lot of people. It’s a really good step in the right direction and I think it’s long overdue. (Case 6, pharmacist) |
| **Burden** | Effort required to engage with Palliate | Emotional strain; perceived workload; sense of responsibility | Reduced service demand; sense of contribution | Physically and mentally and emotionally, it is draining, yes… just being… 24 hours a day, day after day… I gave her a lot of injections… like 70 or 80 injections over time. (Case 16, lay carer, has experience of using Palliate) | It was a little bit time heavy at the start to make sure that the relatives felt confident and sufficiently trained… but then once it was all in place, it saves on at least a number of clinical contact points. (Case 12, GP, involved in recommending Palliate) |
| **Ethicality** | Alignment with personal and professional values | Role conflict; moral discomfort; inequity of responsibility | Compassionate intent; congruence with values | Are we putting people in a difficult position because we're making them carers rather than family? That's why lay care administration is always something that should be voluntary and very, very willing. (Case 6, pharmacist) | It certainly didn't feel like there's any kind of, you know, moral or ethical issues for me. You know, I'm giving care to my dying wife. Seems like a very compassionate thing to do. (Case 16, lay carer, has experience of using Palliate) |
| **Perceived Effectiveness** | Beliefs about achieving intended outcomes | Variable support; inconsistent implementation | Timely symptom management; reduced distress | If you choose your patient and your family carefully, I think they will have a better ending… they’re likely to get more timely symptom control. (Case 19, former national clinical director of a palliative/end-of-life care charity) | I’ve seen it where we’ve turned up to patients late and they’re in agony. And if they’d had somebody else there to give them that, it would definitely mean that people wouldn’t necessarily get to that level. (Case 10, district nurse) |
| **Intervention Coherence** | Understanding of the intervention and its purpose | Information gaps; uncertainty about training | Clear explanations; simple design; training materials | “I think backing the training up with videos is a great idea… that would make life much easier, particularly for older people.” (Case 20, public health professional) | This one is really a no-brainer. You can explain it in two sentences and it's just really obvious what it is. (Case 7, ICB commissioner, has experience commissioning Palliate in their region) |
| **Self-Efficacy** | Confidence in ability to engage | Anxiety about competence; fear of error | Skill acquisition through training; reassurance | I would worry about them being scared about doing that… the amount of people that say to [me], ‘I'm not a nurse. I don't know. I'm not a nurse.’ (Case 26, end-of-life care nurse) | [Talking about a carer she trained]: She picked it up in a couple of hours, was happy, comfortable, competent within a couple of hours of training. (Case 1, pharmacist, has experience of using a variation of Palliate) |
| **Opportunity Costs** | What is given up to engage | Time, social life, emotional energy | Voluntary participation; alignment with caring role | I gave up everything else and just looked after [name] … that was my new job. (Case 13, lay carer, has experience of using Palliate) | It's mainly going to be suited to carers who are there all the time. Otherwise it’s pretty pointless teaching somebody who’s not going to be there when there’s a crisis. (Case 19, former national clinical director of a palliative/end-of-life care charity) |
